# Identification and Pathogenicity Analysis of the Pathogen Causing Spotted Spleen in Muscovy Duck

**DOI:** 10.3389/fvets.2022.846298

**Published:** 2022-05-23

**Authors:** Tianqiao Ke, Dehong Yang, Zhuanqiang Yan, Lijuan Yin, Hanqin Shen, Cuifen Luo, Jingyu Xu, Qingfeng Zhou, Xiaona Wei, Feng Chen

**Affiliations:** ^1^College of Animal Science, South China Agricultural University, Guangzhou, China; ^2^Wen's Group Academy, Wen's Foodstuffs Group Co., Ltd., Xinxing, China

**Keywords:** *Riemerella anatipestifer*, serotypes, spotted spleen, drug sensitivity test, high pathogenicity

## Abstract

Since September 2020, the clinical symptoms of Muscovy duck spleen spots have appeared in Guangdong, Guangxi, Jiangxi, Hunan, Hubei, and other provinces, resulting in a large number of Muscovy duck deaths and great economic losses. The absence of the typical clinical symptoms caused by pathogenic microorganisms makes the cause of the spotted spleen a mystery. High-throughput sequencing results suggested that *Riemerella anatipestifer* (*R. anatipestifer*) may be the pathogen. Then, *R. anatipestifer* was regarded as the research target for isolation, identification, and pathogenicity assessment. After biochemical test, PCR amplification, and serotype determination, it was confirmed that the isolated strain CZG-1 was serotype 15 *R. anatipestifer*. Typical spotted spleen symptoms were observed after CZG-1 infection. Furthermore, drug sensitivity assays showed the similar drug-resistant spectrum of *R. anatipestifer* serotype 15 to other serotypes; for example, all test strains were resistant to polymyxin, gentamicin, and neomycin. The CZG-1 strain has high pathogenicity, and its lethal dose of 50% (LD50) is 35.122 CFU/ml. Virulence gene determination showed that the CZG-1 strain had at least five virulence genes, bioF, TSS9-1, TSS9-2, PncA, and 0373Right. Above all, this study identified and proved that the pathogen of spotted spleen in ducks was *R. anatipestifer* serotype 15, which caused death of ducks without the typical symptoms of bacterial infection. The results of this study enriched the knowledge of symptom after *R. anatipestifer* infection, provided a reference to the identification of the pathogen of spotted spleen, and provided theoretical basis for prevention and control of spotted spleen.

## Introduction

*Riemerella anatipestifer* (*R. anatipestifer*), a Gram-negative bacteria, is a short bacillus that does not form spore and move (1). According to the 16S rRNA gene sequence, *R. anatipestifer* belongs to the rRNA superfamily V *Flavobacterium* family (2). *R. anatipestifer* mainly causes diseases of ducks and geese (the onset age is mainly 3–4 weeks) and can also infect turkeys, other poultry, and wild birds (3). The disease caused by *R. anatipestifer* in ducks is highly pathogenic and infectious, called Rimerellosis anatipestifer infection, also known as infectious serositis of duck, new duck disease, and duck sepsis (4). The main clinical symptoms of *R. anatipestifer* infection are decreased appetite, increased eye and nasal secretions, cough, and dysentery. The most obvious lesion is fibrin exudation on the serosal surface, especially on the pericardium, liver surface, and air sac (3). *R. anatipestifer* infection has caused serious economic losses all over the world (5).

There are many serotypes of *R. anatipestifer*, and the serotypes of infection of the same group of ducks also change constantly (6–12). Until now, at least 21 serotypes of *R. anatipestifer* have been identified (13, 14). Moreover, there is no or less cross-protection between serotypes (14).

Since September 2020, a serious case of high mortality of Muscovy ducks has occurred in Guangdong, Guangxi, Jiangxi, Hunan, Hubei, and other provinces in China. Autopsy showed no obvious symptoms of bacterial infection, but the spleen was covered with white spots, as was the interior of the spleen. At first, it was suspected to be viral infection, but no corresponding nucleic acids of duck hepatitis virus (15), Muscovy duck parvovirus (16), duck enteritis virus (17), duck reovirus (35, 36), and duck tembusu virus (35, 36) were detected in spotted spleen using real-time fluorescent quantitative polymerase chain reaction (RT-qPCR). Therefore, the primary task was to identify the pathogen causing the spotted spleen in ducks.

In this study, *R. anatipestifer* was suspected as the pathogen causing spotted spleen in Muscovy duck according to the results of high-throughput sequencing. Through isolation, identification, serotype test, and pathogenicity test, we identified that the pathogen was *R. anatipestifer* serotype 15. Furthermore, drug-sensitivity and virulence genes of isolated strains have been detected. The results of this study will provide a theoretical basis for the pathogen identification and the prevention of spotted spleen in Muscovy duck.

## Materials and Methods

### High-Throughput Sequencing

Briefly, total RNA was extracted from spotted spleen using TRIzol Reagent (Invitrogen, USA), followed by an RNase free DNase I (Qiagen, Hilden, Germany) treatment to remove any DNA contamination, then double-stranded cDNA synthesis was performed by using Maxima™ H Minus Double-Stranded cDNA Synthesis Kit (Thermo Scientific, USA), both according to the manufacturer's protocols. In addition, the double-stranded cDNA was purified by a 1:1 ratio of AMPure XP beads (Beckman, USA), and quantified using a Qubit dsDNA HS Assay kit (Thermo Fisher, USA), following the manufacturer's instructions. A total of 300 ng of cDNA was input to the ligation sequencing kit (Oxford Nanopore Technologies, UK), and barcoded using the native barcoding expansion kit (Oxford Nanopore Technologies, UK) according to the manufacturer's protocols. The library was sequenced on the MinION Mk1B instrument (Oxford Nanopore Technologies, UK). *De novo* assembly was used for sequence assembly, under the App Map function of CLC Genomics Workbench version 21.0.4 (Qiagen, Germany) with default parameters as previously described (18, 19).

### Isolation and Identification of *R. anatipestifer*

The spotted spleens and livers of dead ducks were collected and treated in a clean bench to avoid contamination. The internal tissues were collected using sterile inoculating loop and spread on TSA (Tryptone soy agar) plates with 5% fetal bovine serum (Solarbio, China). The plates were placed at 37°C for 18–24 h with 5% CO_2_. The single colonies were selected and inoculated into TSB (Tryptic soy broth) medium containing 5% fetal bovine serum, and cultured in 37°C for 12–16 h.

The gram staining assay was carried out with Gram Stain Kit (Solaibao, China) as per the manufacturer's instruction. The bacterial morphology and staining characteristics were observed under a microscope with a 100 × oil immersion lens (20).

To make sure that the isolated strains were *R. anatipestifer*, PCR amplification was performed with the two specific primers GYRB and 190 F/843 R (Supplementary Table 1). Briefly, bacteria solutions were heated at 100°C for 5 min and centrifuged at 8,000–9,000 rpm/min for 5 min, the supernatants containing bacterial genome were collected as the DNA template. The PCR reaction system consisted of 10 μl of 2 × Taq Mix enzyme, 1 μl of each primer, 1 μl of template, and 5 μl of ddH_2_O, with the following procedures: 94°C pre-denaturation for 3 min, 94°C denaturation for 30 s, 55°C annealing for 30 s, 72°C extension for 1 min, a total of 35 cycles, then extension at 72°C for 10 min, and holding at 4°C.

Biochemical test was carried out with a Bacterial Microbiochemical Reaction Tube (Hangzhou Microbial Reagent Co., Ltd., China) as per the manufacturer's instructions. Briefly, isolated strains were inoculated into different biochemical reaction tubes in different ways according to the instructions and then cultured at 37°C for 24–48 h.

After isolation and identification, one of the *R. anatipestifer* strains isolated from spotted spleen was named CZG-1, which was used in further studies.

### Pathogenicity Test

To identify the pathogenicity, CZG-1 was diluted into different concentrations, 1 × 10^4^, 1 × 10^3^, 1 × 10^2^, 80, 60, 40, 20, and 10 CFU/ml, to infect Muscovy ducks. A total of 90 14-day-old Muscovy ducks were randomly assigned into nine groups, 10 in each group. Ducks in 8 groups were infected with 0.5 ml of different concentrations of CZG-1, respectively, *via* intramuscular injection of the leg, and the remaining groups of ducks were inoculated with 0.5 ml of PBS as a mock-infection control. The death of ducks in each group was recorded every day. The lethal dose of 50% (LD_50_) of CZG-1 was calculated by the Bliss method (21). The dead ducks were autopsied and the tissue lesions were collected for pathological examination. This study was approved by the Animal Care Committee of South China Agricultural University (approval ID: SYXK-2019-0136). All study procedures and animal care activities were conducted per the recommendations in the Guide for the Care and Use of Laboratory Animals of the Ministry of Science and Technology of the People's Republic of China.

### Serotype, Drug Sensitivity, and Virulence Gene Detection

After purification and identification, the CZG-1 strain was washed three times with PBS (phosphate buffer saline, pH 7.4), and the bacterial suspension was subjected to 121°C for 2 h. After centrifugation at 7,000–9,000 rpm/min for 10 min, the supernatants were collected and used as the heat-stable antigen for serological identification by agar gel precipitin test (14). Briefly, the agar plates containing 10% NaCl were punched with several holes using a puncher, six holes to form a circle, and then a hole was punched in the middle of the circle. Heat-stable antigen (20 μl) was added to the middle hole, and 20 μl of different immune serums was added to the surrounding holes, respectively. Plates were placed at 37°C with 5% CO_2_ for 24 h to observe the antigen–antibody complex precipitation band (22).

Drug sensitivity analysis was carried out with disc diffusion test. The susceptibility papers (Hangzhou Microbial Reagent Co., Ltd., China) were used as per the manufacturer's instruction. Briefly, the tested strains were spread evenly on TSA plates and then the susceptibility papers were stuck evenly on the plate. The TSA plates were cultured at 37°C with 5% CO_2_ for 18 h and the sensitivity of *R. anatipestifer* strains to different drugs were determined by measuring the size of the inhibition zone.

Virulence gene determination was carried out by PCR amplification. The detected virulence genes were LPS-1, vapD, bioF, TSS9-1, TSS9-2, PncA, and 03730Right (23–29). The specific primers used in this study are listed in Supplementary Table 1. The PCR procedure was the same as mentioned above.

## Results

### Histopathological Sections of Clinically Dead Ducks

**Figure 1:**
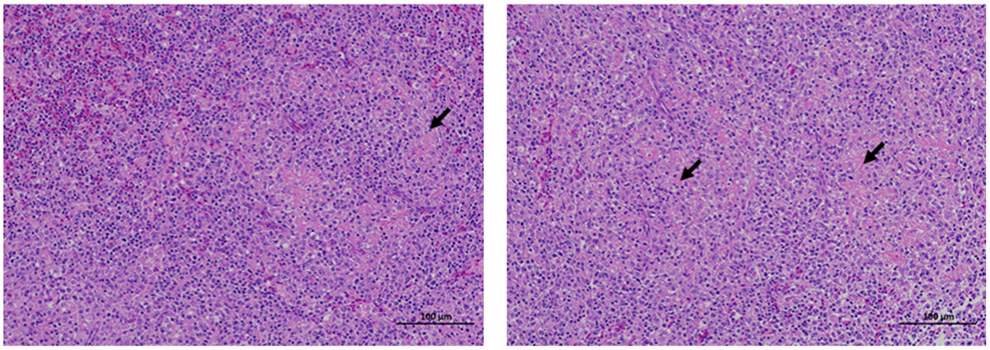
Histopathological section results of tissues collected from clinically dead ducks. The tissues were fixed with 10% formalin, made into slices, HE stained, and observed under an optical microscope. The arrow shows the cells' loss of cellular structure to eosinophilic homogeneous fibrous material in the spleen. The scale means 100 μm.

The dead ducks were collected and autopsied. There were no obvious pathological changes in the heart, liver, brain, and other tissues of the dead ducks, but the spleen showed white spots. Spotted spleen was collected for histopathological analysis. The results are shown in Figure 1; the boundary between the red and white pulp was not clear, and the white pulp was diffused throughout the spleen. Small areas of fibrinoid necrosis were seen in the white pulp, and the cell structure disappeared into eosinophilic homogeneous fibrous material. No obvious inflammatory reaction was observed.

### High-Throughput Sequencing Analysis

Six spotted spleens from different dead ducks from different provinces were selected to high-throughput sequencing. The sequences obtained were then blasted *via* NCBI (National Center of Biotechnology Information). The blast results are shown in Table 1; *R. anatipestifer* was blasted in all of the six samples with the highest similarity, and then Muscovy duck parvovirus was blasted in four samples and Goose parvovirus was blasted in two samples. Considering the highest detection rate and similarity, we targeted *R. anatipestifer* as the major pathogen of spotted spleen for further study, including isolation, identification, and pathogenicity assessment.

**Table 1:** Blast results after high-throughput sequencing of six spotted spleen samples.

	***R. anatipestifer***	**Muscovy duck parvovirus**	**Goose parvovirvs**	**Duck parvovirus**	***Chryseobacterium***	***Pedobacter cryoconitis***	**Barbarie duck parvovirus**
Samples 1	1–15	16, 18–19, 20, 22, 25, 28–30	17	21, 23–24, 26–27			
Samples 2	1–14	17, 19–20, 23, 28, 30	24	21–22, 25–27, 29	15	16	18
Samples 3	1–14	15–17, 20–22, 24, 28–30	19, 27	18, 23, 25–26			
Samples 4	1–14	16, 21–23, 27, 29–30	15, 17–19	20, 24–26, 28			
Samples 5	1–14	19, 22–25, 29–30	17	18, 21, 26–28	15	16	20
Samples 6	1–14	16–19, 22, 24, 28–30	20	21, 23, 25–27			15

*At least seven pathogenic microorganisms were blasted and only listed the top seven in this table. The numbers in the table indicate the order of different microorganisms in different samples. R. anatipestifer was listed as the top 14 in all samples blast results. The numbers in the table indicate the order in which the blast results appear and only the top 30 are listed*.

### Isolation and Identification of *R. anatipestifer*

After being cultured on a TSA plate, the round, swell, neat edge, and milky white single colonies with a smooth surface and a diameter of 1–2 mm were obtained, named CZG-1. The Gram staining showed that the CZG-1 strain was Gram-negative bacteria with a red rod shape. PCR amplification with two different specific primer pairs showed that there were two different bands with sizes of 644 and 194 bp, respectively, as shown in Supplementary Figure 1. Biochemical test results showed that the CZG-1 strain could use oxidase, urease, and contact enzyme; could not use carbohydrates, citrate, urea, arginase, and lysine enzyme; and could not produce H_2_S and indole (Table 2). Taken together, isolated strain from spotted spleen named CZG-1 was *R. anatipestifer*. In serological typing assay, a clear white band appeared between the heat-stable antigen hole and the No. 15 immune serum hole (data not shown), which suggested that the CZG-1 strain was the *R. anatipestifer* serotype 15.

**Table 2:** The results of biochemical test of isolated strains.

**Project**	**Result**	**Project**	**Result**	**Project**	**Result**	**Project**	**Result**
Glucose	−	Arabic candy	−	Hydrogen sulfide	−	Mannose	−
Sucrose	−	Trehalose	−	The urea	−	Sorbitol	−
Maltose	−	Sorbose	−	M.R	−	Dulcitol	−
Starch	−	Inositol	−	V.P	−	Lysine enzyme	−
Lactose	−	Mannitol	−	Arginase	−	Contact enzyme	+
Galactose	−	Nitrate reduction	−	Indole	−	Oxidase	+
Erythrose	−	Citrate	−	Fructose	−	Urease	+

*“−” means negative result of biochemical test; “+” means positive result of biochemical test*.

### Pathogenicity Test of CZG-1

**Figure 2:**
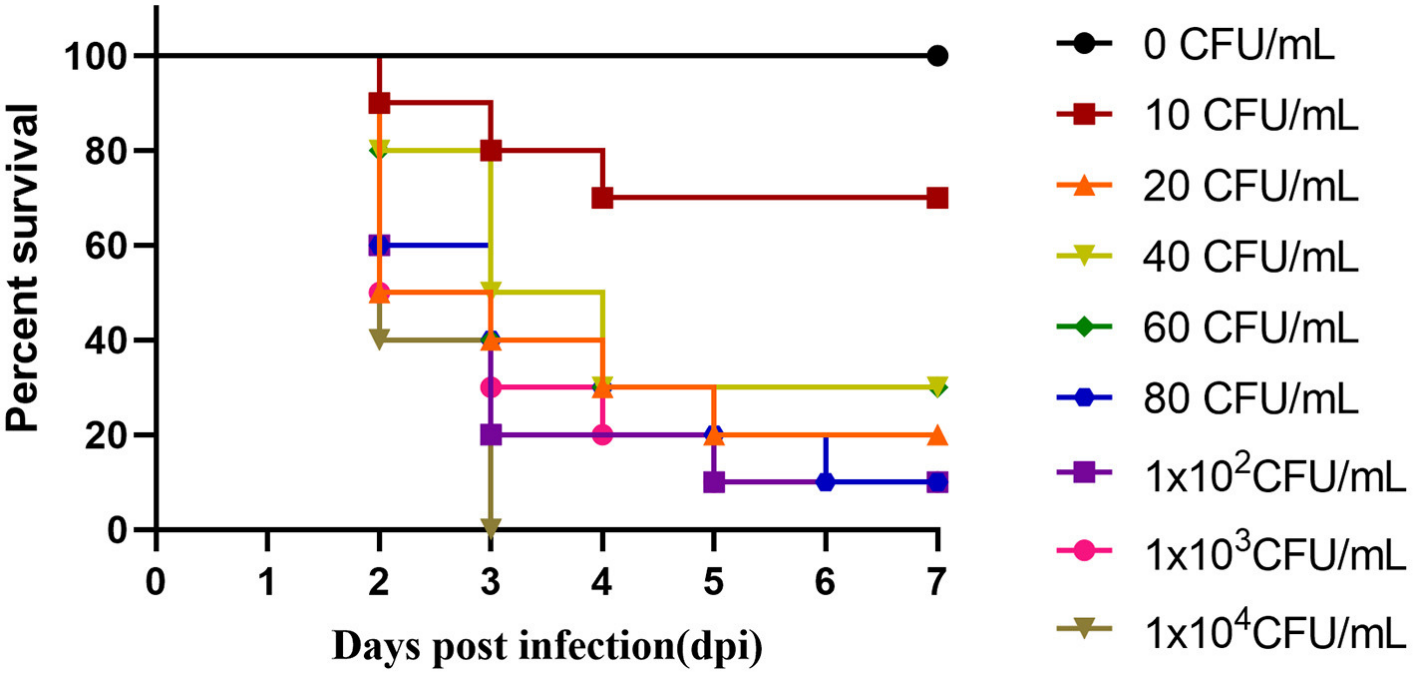
Survival curves of Muscovy duck in pathogenicity test. The horizontal axis represents days after infection, the vertical axis represents survival, and the different curves represent different dose groups.

The 14-day-old Muscovy ducks were used to detect LD_50_ of the CZG-1 strain. Eight groups of ducks were infected with different concentrations of CZG-1, and one group of ducks was inoculated with medium as mock-infection control. As shown in Figure 2, ducks in infected groups began to die on the 2nd day after infection. All of the ducks infected with 1 × 10^4^ CFU/ml CZG-1 died on the 3rd day after infection. After 1 week of continuous observation, the number of remaining ducks in each group was different. The remaining ducks infected with 10 CFU/ml CZG-1 was 7, and the survival rate was 70%. At most three ducks were left after inoculation with other concentrations of CZG-1, and even the infection dose was 20 CFU/ml. No ducks died in the mock-infected group (Figure 2). By calculation, the LD_50_ of CZG-1 was 35.122 CFU/ml.

**Figure 3:**
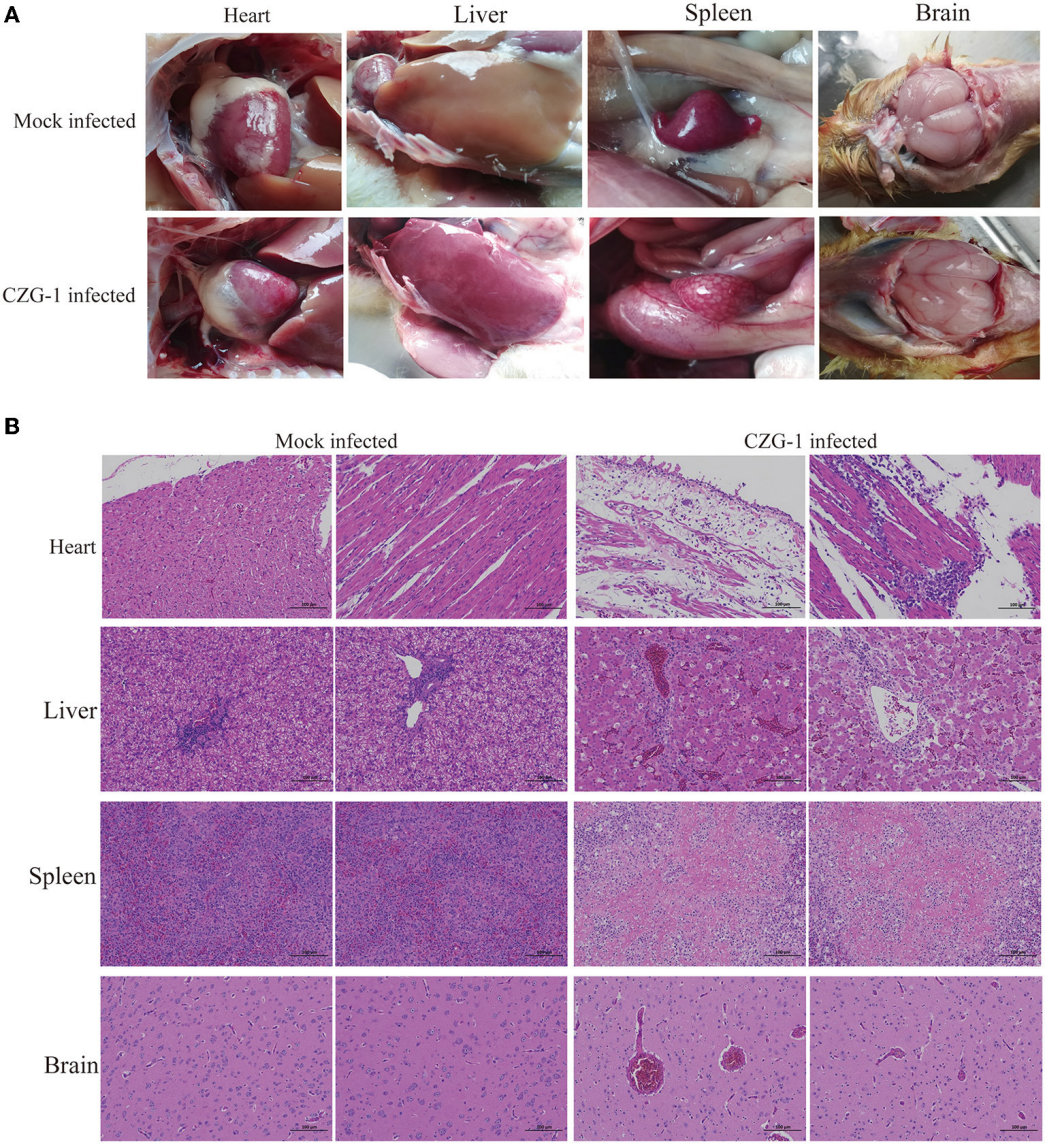
Autopsy and pathological sections of tissues after CZG-1 infection. **(A)** Autopsy results of normal and dead ducks infected with CZG-1 strain. **(B)** Pathological section results of tissues collected from normal and dead ducks.

Necropsy of the dead duck revealed a typical spotted spleen with mild pericarditis, liver capsule, hepatorrhagia, and cerebral hemorrhage (Figure 3A), which proved that the CZG-1 strain can cause spotted spleen in Muscovy ducks. Histopathological sections of the spotted spleen (Figure 3B) showed extensive white myeloid fibrinoid necrosis, with a large loss of cell structure, or residual necrotic fragmented nuclei. The necrotic focus was a large area of eosinophilic homogeneous fibrinoid material. There was no obvious inflammatory reaction in spotted spleen. Histopathological sections of the dead duck heart showed a wide range of epicardial edema, surrounding myocardial edema, and loose arrangement of fibers, accompanied by a small amount of inflammatory cell exudation, epicardial necrosis, necrotic fiber eosinophilic enhancement and detachment, and a small number of inflammatory cell focal infiltration in local myocardial interstitium (Figure 3B). The liver sections of dead ducks showed many punctured necrosis of liver cells, a large number of sinus congestion, and a large number of inflammatory cells in the portal area (Figure 3B). Pathological sections of the brain of dead ducks showed only a small amount of extravasation of blood vessels (Figure 3B).

### Drug Sensitivity and Virulence Gene Detection of CZG-1

**Figure 4:**
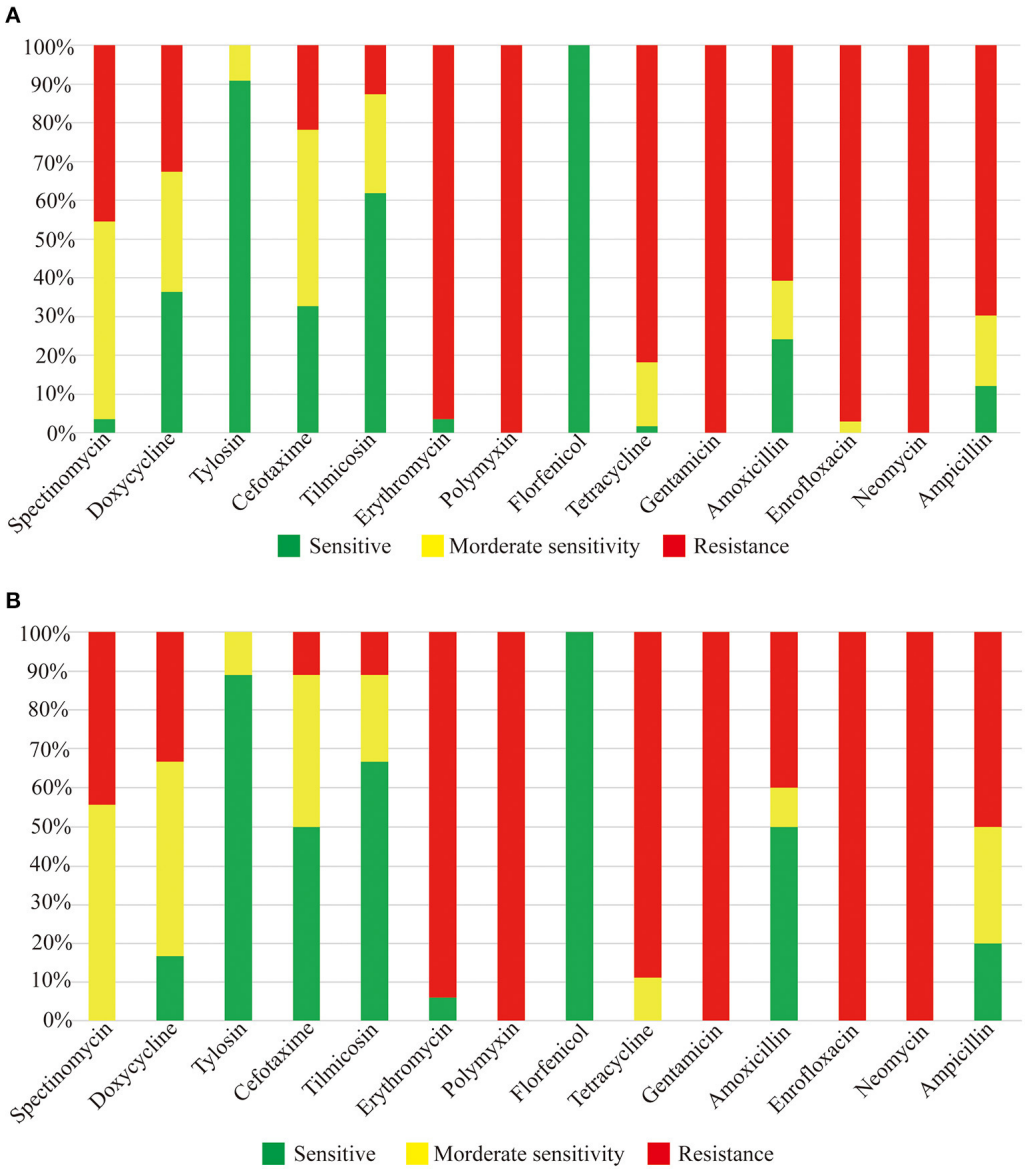
Drug sensitivity test results. **(A)** Drug sensitivity results of serotype 15 *R. anatipestifer* strains. **(B)** Drug sensitivity results of other serotype strains.

Eighteen strains of *R. anatipestifer* serotype 15 were selected to test the sensitivity to 14 drugs, including Spectinomycin, Doxycycline, Tylosin, Cefotaxime, Tilmicosin, Erythromycin, Polymyxin, Florfenicol, Tetracycline, Gentamicin, Amoxicillin, Enrofloxacin, Neomycin, and Ampicillin. The results are shown in Figure 4A; serotype 15 strains of *R. anatipestifer* were resistant to Polymyxin, Gentamicin, Neomycin, Erythromycin, and Enrofloxacin, and were sensitive to Florfenicol and Tylosin; different strains have different sensitivities. Besides, 18 strains of serotype 15, 10 strains of serotype 1, 16 strains of serotype 3, six strains of serotype 5, and three strains of serotype 6 also detected the drug sensitivity. As shown in Figure 4B, the tested strains have similar drug resistance spectrums to serotype 15 strains.

**Figure 5:**
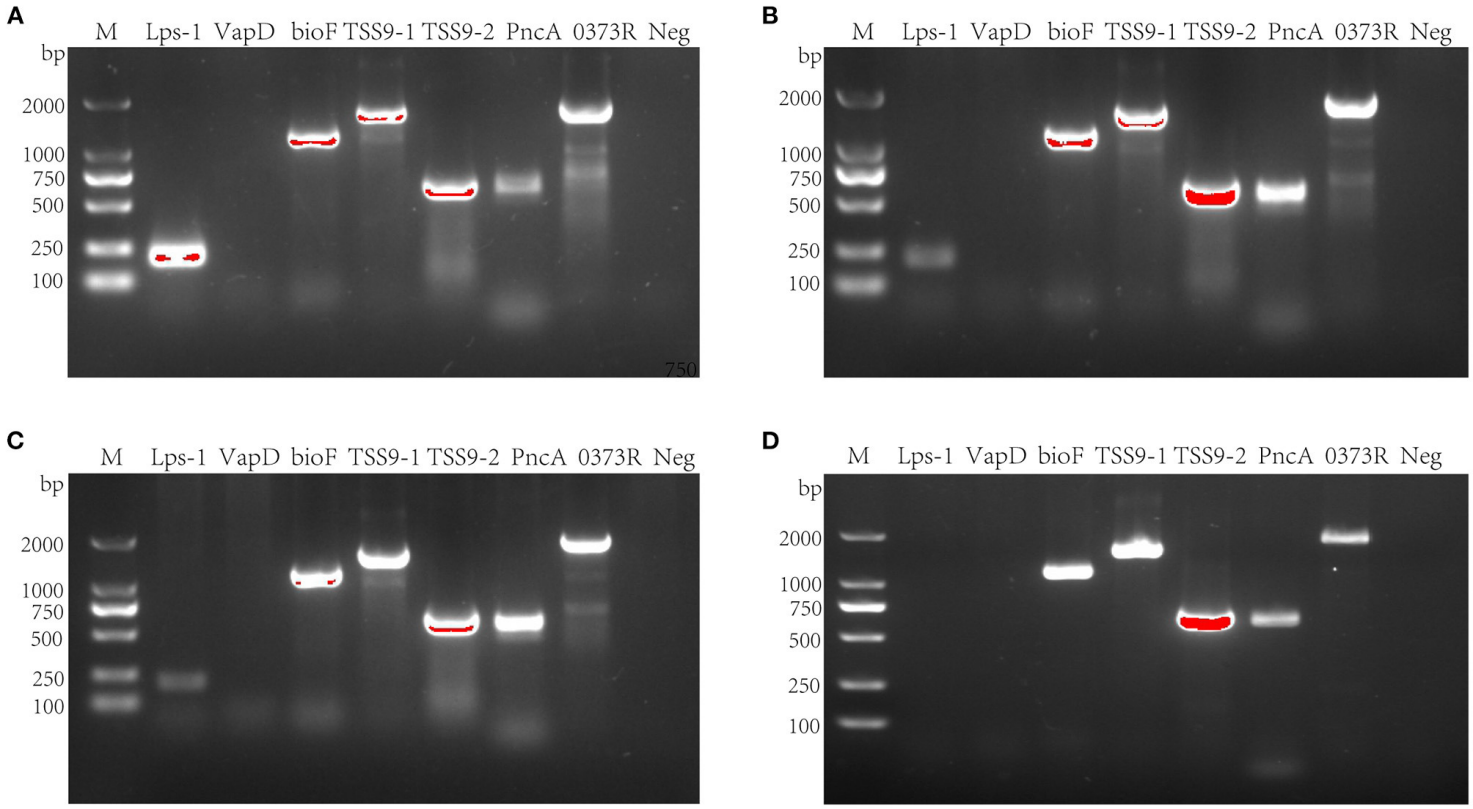
Virulence gene detection of *R. anatipestifer*. **(A)** Virulence gene detection of serotype 1 *R. anatipestifer*. **(B)** Virulence gene detection of serotype 3 *R. anatipestifer*. **(C)** Virulence gene detection of serotype 5 *R. anatipestifer*. **(D)** Virulence gene detection of serotype 15 *R. anatipestifer*. M means DNA marker; 0373R means 0373Reight gene; Neg means negative control.

To detect the virulence genes, bioF, TSS9-1, TSS9-2, PncA, and 0373Right, LPS-1, and vapD were chosen as test targets (Figure 5). After PCR amplification, 55 strains of serotype 15 *R. anatipestifer* had the five virulence genes, bioF, TSS9-1, TSS9-2, PncA, and 0373Right, and did not have the LPS-1 and vapD genes (Figure 5D). Besides, virulence genes were also tested on 19 strains of serotype 1, 35 strains of serotype 3, and 26 strains of serotype 5. Serotypes 1, 3, and 5 *R. anatipestifer* strains contained LPS-1, bioF, TSS9-1, TSS9-2, PncA, and 0373Right, but not vapD (Figures 5A–C).

## Discussion

Since September 2020, ducks with spotted spleen have occurred frequently in Guangdong, Guangxi, Jiangxi, Hunan, and Hubei provinces of China, resulting in a large number of duck deaths and causing great economic losses. The absence of the typical clinical symptoms caused by bacteria and undetectable conventional viral nucleic acids make the cause of the spotted spleen a mystery. The purpose of this study was to identify the pathogen of spotted spleen.

Due to the uncertainty of which pathogen caused the symptoms of spotted spleen, the tissue samples of infected ducks in different regions were collected and high-throughput sequenced. The results showed that all the tissue samples contained *R. anatipestifer* with the highest similarity, so the next step was to verify whether spotted spleen are factually caused by *R. anatipestifer*. Through bacterial isolation, Gram staining, PCR identification, biochemical determination, and animal pathogenicity test, the pathogen was finally identified as *R. anatipestifer*, and to be specific, serotype 15.

In the results of high-throughput sequencing (Table 1), we can see that *R. anatipestifer* had the highest similarity rate after being blasted in all of the six samples. Besides, Muscovy duck parvovirus, Duck parvovirus, and Goose parvovirus were also blasted, and whether parvovirus was one of the pathogens causing the spotted spleen needs further study; in this study, we just focused on *R. anatipestifer*.

Through serotype identification, the isolated strain of CZG-1 belongs to serotype 15. The LD_50_ of CZG-1 was 35.122 CFU/ml. In Dou et al. results about *R. anatipestifer*, the LD_50_ of serotype 1 CH3 strain was 7.50 × 10^7^ CFU/ml (30). Another serotype 1 *R. anatipestifer* YL4 strain has an LD_50_ of about 4.74 × 10^6^ CFU/ml (31). Besides, the LD_50_ of serotype 2 *R. anatipestifer* Yb2 strain (31), Th4 strain (32), and WZX01 strain (33) was 1.07 × 10^5^, 4.41 × 10^8^, and 4.20 × 10^3^ CFU/ml, respectively. HXb2 strain (31, 34), serotype 10, has an LD50 of 82 CFU/ml. After comparison, we could find that the newly discovered CZG-1 strain that could cause spotted spleen had a higher pathogenicity, which may explain why ducks died without typical clinical symptoms caused by *R. anatipestifer*.

Drug sensitivity test can guide the use of drugs in the clinic and control pathogenic bacteria. In this study, we determined and compared the drug resistance of 18 strains of serotype 15, 10 strains of serotype 1, 16 strains of serotype 3, six strains of serotype 5, and three strains of serotype 6 of *R. anatipestifer*. As shown in Figure 3, these strains have a similar drug-resistant spectrum, which means one sensitive drug can control or inhibit various serotypes of *R. anatipestifer*, which has important clinical guiding significance.

After testing for LPS-1, vapD, bioF, TSS9-1, TSS9-2, PncA, and 0373Right virulence genes (23–29) of serotype 15 *R. anatipestifer*, only LPS-1 and vapD were found to be absent. The detection of these seven virulence genes of serotype 1, serotype 3, and serotype 5 *R. anatipestifer* showed that these three serotypes of *R. anatipestifer* did not contain only the vapD. This is the difference between serotype 15 *R. anatipestifer* and the other three serotypes. Although there are limitations, it can be used as a theoretical reference for further study of serotype 15 *R. anatipestifer*. However, whether the five virulence genes are related to the high pathogenicity of the strain CZG-1 still needs further study.

A significant result of this study was the discovery of a pathogen causing spotted spleen in Muscovy duck, which was *R. anatipestifer* serotype 15. The symptoms associated with *R. anatipestifer* infection have been rarely reported internationally. The results of this study will provide a theoretical basis for the identification of the pathogen of spotted spleen in ducks, and provide a reference for a follow-up in-depth study on the mechanism of this symptoms.

## Data Availability Statement

The original contributions presented in the study are included in the article/Supplementary Material, further inquiries can be directed to the corresponding authors.

## Ethics Statement

The animal study was reviewed and approved by the Institutional Animal Care and Use Committee of South China Agricultural University. Written informed consent was obtained from the owners for the participation of their animals in this study.

## Author Contributions

FC designed and supervised the progress of the study. TK, DY, LY, and CL completed most of the work of this study. XW and ZY wrote the manuscript and QZ and FC modified it. All authors contributed to the article and approved the submitted version.

## Funding

This work was supported by the Guangdong Basic and Applied Basic Research Foundation (2019A1515012006) and the Key Research and Development Program of Guangdong Province (2020B020222001).

## Conflict of Interest

DY, ZY, LY, HS, CL, JX, QZ, and XW was employed by company Wen's Foodstuffs Group Co., Ltd. The remaining authors declare that the research was conducted in the absence of any commercial or financial relationships that could be construed as a potential conflict of interest.

## Publisher's Note

All claims expressed in this article are solely those of the authors and do not necessarily represent those of their affiliated organizations, or those of the publisher, the editors and the reviewers. Any product that may be evaluated in this article, or claim that may be made by its manufacturer, is not guaranteed or endorsed by the publisher.

## Supplementary Material

Supplementary Figure 1 The results of identification of isolated strains. **(A)** Gram strain results of isolated strains. **(B)** PCR identification of isolated strains. M means DNA marker; NC means negative control; PC means positive control; RA means identified *R.anatipestifer*.

Supplementary Table 1 Primers used in this study.
