# Supplementary figures and images for: Identification and Pathogenicity Analysis of the Pathogen Causing Spotted Spleen in Muscovy Duck
Source: Front Vet Sci. 2022 May 23;9:846298. doi: 10.3389/fvets.2022.846298 (PMC9169529; doi:10.3389/fvets.2022.846298)

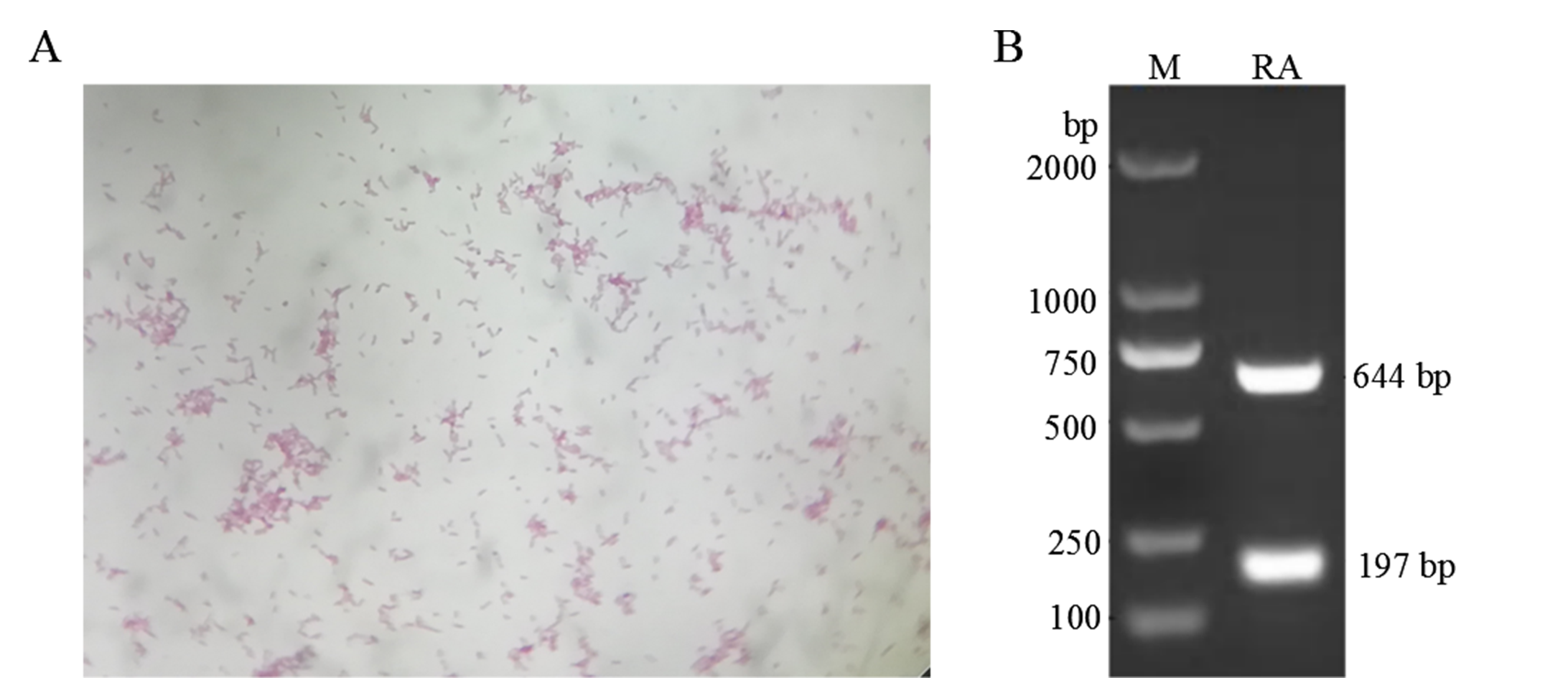

Supplement: Supplementary Figure 1 — The results of identification of isolated strains. (A) Gram strain results of isolated strains. (B) PCR identification of isolated strains. M means DNA marker; NC means negative control; PC means positive control; RA means identified R.anatipestifer. [file Image_1.TIF]
